# The Bright and Dark Sides of Nitric Oxide in Neurodegenerative Diseases

**DOI:** 10.3390/jpm16050246

**Published:** 2026-05-01

**Authors:** Lucia Buccarello, Costanza Montagna, Sabina Di Matteo, Renata Mangione, Giuseppe Carota, Jay Sibbitts, Romana Jarosova, Susan M. Lunte, Giacomo Lazzarino, Giuseppe Caruso

**Affiliations:** 1 Departmental Faculty of Medicine, UniCamillus-Saint Camillus International University of Health and Medical Sciences, 00131 Rome, Italy; lucia.buccarello@unicamillus.org (L.B.); costanza.montagna@unicamillus.org (C.M.); sabina.dimatteo@unicamillus.org (S.D.M.); renata.mangione@unicamillus.org (R.M.); giacomo.lazzarino@unicamillus.org (G.L.); 2 IRCCS San Camillo Hospital, 30126 Venice, Italy; 3 Department of Biomedical and Biotechnological Sciences, University of Catania, 95123 Catania, Italy; giuseppe-carota@outlook.it; 4 Department of Physical Sciences, Truman State University, Kirksville, MO 63501, USA; jsibbitts@truman.edu; 5 Department of Chemistry, Colorado State University, Fort Collins, CO 80523, USA; romana.jarosova@colostate.edu; 6 Department of Chemistry, University of Kansas, Lawrence, KS 66045, USA; slunte@ku.edu; 7 Ralph N. Adams Institute for Bioanalytical Chemistry, University of Kansas, Lawrence, KS 66045, USA; 8 Department of Pharmaceutical Chemistry, University of Kansas, Lawrence, KS 66045, USA

**Keywords:** neurodegeneration, oxidative stress, nitric oxide, neurotoxicity, neuroprotection, carnosine

## Abstract

Nitric oxide (NO) plays an important role in neuronal communication, synaptic plasticity and vascular regulation. Due to its important function in neuronal homeostasis, NO imbalance is associated with neurodegeneration. Specifically, in Alzheimer’s disease (AD), amyotrophic lateral sclerosis (ALS), Parkinson’s disease (PD) and frontotemporal lobar degeneration (FTLD), an excessive amount of NO, mostly produced by inducible NO synthase (iNOS), reacts with superoxide to form peroxynitrite, driving oxidative/nitrosative stress, mitochondrial dysfunction, and aberrant protein modifications. In AD, NO dysregulation promotes amyloid-β (Aβ) accumulation, tau hyperphosphorylation and synaptic loss, creating a self-perpetuating cycle of neuronal damage. NO’s dual role, protective at physiological levels but harmful if overproduced, underscores the therapeutic potential of antioxidant compounds that restore the balance of NO/NOS (especially iNOS) while preserving physiological functions. However, despite the emerging role of antioxidant-based therapeutic approaches, clinical translation is limited by the complexity of NO signaling and the absence of safe, specific NOS inhibitors. By targeting the molecular switch from protective to toxic, NO activity may offer new personalized treatment avenues for neurodegenerative diseases.

## 1. Introduction

Nitric oxide (NO) is a freely diffusible gaseous free radical molecule involved in a wide array of physiological processes [1]. It was the first gas molecule discovered to function as a second messenger in mammals. The first biological role of NO was as a vasodilator, and it was originally known as “endothelium-derived relaxing factor”, or “EDRF”, reflecting its involvement in blood vessel tone control; the mechanism of action underlying this physiological function is related to the activation of soluble guanylyl cyclase (sGC) through direct binding to heme iron (Fe-nitrosylation), and the induction of cyclic GMP (cGMP)-mediated signaling [2,3]. Eventually, NO’s participation in immune response, neurotransmission, and many other biological functions was recognized, establishing the key role of this molecule in cellular and physiological signaling [4,5]. NO is biologically generated by NO synthases (NOSs), a class of NADPH-dependent enzymes that use L-arginine and molecular oxygen as substrates [6]. In mammals, three major nitric oxide synthase (NOS) isoforms have been identified: neuronal NOS (nNOS) and endothelial NOS (eNOS), both constitutively active, and the cytokine-dependent inducible NOS (iNOS), which is predominantly expressed by immune cells in response to pathogenic challenge.

NO’s activation of sGC is considered the “canonical” mechanism of NO signaling [7], and it represents a clear example of paracrine signaling. NO, produced by NOS, diffuses into target cells and binds to the heme group of sGC, inducing a conformational change that activates the enzyme [8]. Activated sGC catalyzes the conversion of GTP to cGMP, a second messenger that regulates downstream effectors, including protein kinases (e.g., protein kinase G (PKG)), ion channels, and phosphodiesterases. PKG is a key mediator of NO–cGMP signaling, and its dysregulation has been implicated in cardiovascular diseases, such as hypertension and heart failure due to impaired vasodilation. This pathway underlies key physiological effects of NO, such as vasodilation, inhibition of platelet aggregation, and neurotransmission [9,10,11,12,13]. Other “non-classical” NO signaling mechanisms, primarily covalent post-translational protein modification by a series of reactive nitrogen species (RNS) derived from the reaction of NO with other small molecules, including oxygen free radicals, have been extensively described [14].

Among these post-translational modifications, tyrosine nitration occurs under conditions of NO overproduction and mostly depends on the reaction of proteins with peroxynitrite (ONOO^−^), a well-known dangerous NO-derived RNS, which is generated by the reaction of NO with other oxygen-derived radical and nonradical species. Nitration occurs through a covalent addition of a nitro group (-NO_2_) to the phenolic ring of tyrosine residues [15]. Since tyrosine nitration irreversibly affects protein structure and function, it is commonly classified as a damaging modification rather than signaling; therefore, this phenomenon is commonly known as nitrosative (or nitroxidative) stress [15] and often results in detrimental effects, such as protein loss-of-function and DNA damage [16,17].

On the other hand, S-nitrosylation is a redox-reversible post-translational modification driven by the covalent addition of an NO moiety to thiol (-SH) containing molecules. SH-containing molecules can be both low-molecular-weight thiols, such as cysteine, glutathione (GSH), and coenzyme-A (CoA), and high-molecular-weight thiols such as proteins. The covalent addition of an NO moiety converts the molecules into a S-nitrosothiol (SNO) derivative, such as S-nitrosylated cysteine (SNO-Cys), S-nitrosoglutathione (GSNO), S-nitroso-coenzyme-A (SNO-CoA), and S-nitrosylated proteins (PSNOs).

Thousands of proteins can be S-nitrosylated, influencing distinct physiological and pathological processes [18,19,20]. As for the other PTMs, reversibility is achieved via enzymatic systems called de-nitrosylases, such as thioredoxin (Trx)/thioredoxin reductase (TrxR), GSNO/GSNO reductase (GSNOR) [21,22], and SNO-CoA/SNO-CoA reductase (ScoR) couples, which can reduce SNOs back to the sulfhydryl state (SH) [22,23]. Throughout all the different mechanisms of action, NO plays an essential role as a signaling molecule in the central nervous system (CNS) and is involved in neurotransmission, neurovascular regulation, synaptic plasticity, and neuroprotection. Despite this, when it is dysregulated, it contributes to the pathogenesis of neurodegeneration [24,25,26]. Specifically, within the CNS, the neuroprotective and neurotoxic double role of NO is determined by factors such as concentration, duration of exposure, and cellular context [27,28,29]. It plays essential roles in modulating neuronal communication, memory formation, sexually dimorphic behaviors, and neuroendocrine regulation, making it a potential target for therapeutic intervention.

Notably, dysregulation of NO signaling has been implicated in the pathogenesis of several neurodegenerative disorders, including Alzheimer’s disease (AD), Parkinson’s disease (PD), and frontotemporal dementia (FTD) [19,29,30,31]. Interestingly, despite their distinct genetic and molecular origins, these diseases all exhibit the commonality of excessive NO production and nitrosative stress [32,33,34,35], contributing to progressive neuronal degeneration, injury, and cell death.

A key pathological mechanism underlying NO-induced neurotoxicity involves the disruption of redox homeostasis, particularly by causing an imbalance between reactive oxygen species (ROS) and RNS [36,37,38]. Excessive NO can react with superoxide anions (O_2_·^−^) to form ONOO^−^, a highly reactive oxidant that damages proteins, lipids, and DNA. Furthermore, S-nitrosylation, a redox-dependent post-translational modification mediated by NO, can alter protein function and contribute to synaptic dysfunction and neuronal loss.

In this narrative review, based on recent and pivotal research relevant to the field, we aimed to highlight the complex role of NO signaling in neurodegenerative diseases, focusing on its involvement in oxidative and nitrosative stress dysregulation and discussing its relevance for the development of targeted and personalized therapeutic strategies.

## 2. Nitric Oxide Biosynthesis and Regulation in the Central Nervous System

NO is produced by three main NOS isoforms: constitutive calcium-dependent neuronal (nNOS or NOS1), endothelial (eNOS or NOS3), and inducible (iNOS or NOS2) isoforms. All NOS isoforms are homodimeric heme-containing enzymes, and each contains an N-terminal oxygenase domain linked via a calmodulin (CaM)-binding sequence to the C-terminal reductase domain [39]. The enzymatic reaction catalyzed by NOSs produces NO and L-citrulline from L-arginine, molecular oxygen (O_2_) and NADPH. This process requires different cofactors, which include tetrahydrobiopterin (BH4), NADPH, heme, flavin adenine dinucleotide (FAD), and flavin mononucleotide (FMN) [40].

Although all three are present in the CNS, nNOS is the principal source of neuronal NO under physiological conditions [41]. eNOS is predominantly expressed in endothelial cells and contributes to neurovascularization and cerebral blood flow [42], while iNOS is physiologically absent in the CNS, but can be induced in glia and immune cells during inflammation, producing large amounts of NO [43].

nNOS constitutes the predominant source of NO in neurons and is localized to synaptic spines, and its expression is tightly regulated at multiple transcriptional and post-transcriptional levels, and through protein–protein interactions. In particular, nNOS is regulated transcriptionally by transcription factors such as Sp1 and CREB that modulate its expression in response to neuronal activity. CREB is a central regulator of activity-dependent gene expression, and its dysregulation has been linked to neurological disorders through impaired neuronal survival and synaptic plasticity. In neurons, NO synthesis is tightly linked to glutamatergic signaling. Activation of synaptic glutamate-induced activation of NMDA (N-methyl-D-aspartate) receptors (NMDARs) [40] under physiological conditions results in moderate Ca^2+^ influx, stimulating nNOS and generating NO at levels that support synaptic plasticity, including long-term potentiation (LTP), neuronal survival, and CREB activation [44]. NMDAR dysfunction, particularly the shift from synaptic to extra-synaptic signaling, is strongly involved in neurodegenerative diseases such as AD through the promotion of excitotoxic pathways. However, pathological conditions such as excessive glutamate release or amyloid-β (Aβ) accumulation promote hyperactivation of extra-synaptic NMDARs. This leads to excessive NO production and ROS generation, contributing to excitotoxicity and neurodegeneration [45].

Importantly, the differential impact of synaptic versus extra-synaptic NMDAR activation on NO production presents therapeutic implications. Memantine, an NMDA receptor antagonist approved by the FDA, preferentially inhibits extra-synaptic NMDARs and has demonstrated efficacy in mitigating symptoms of AD and dementia with Lewy bodies [46,47,48]. These findings underscore the significance of the NMDAR-NO axis in neurodegenerative disease pathogenesis.

## 3. The Double Face of Nitric Oxide

**Figure 1:**
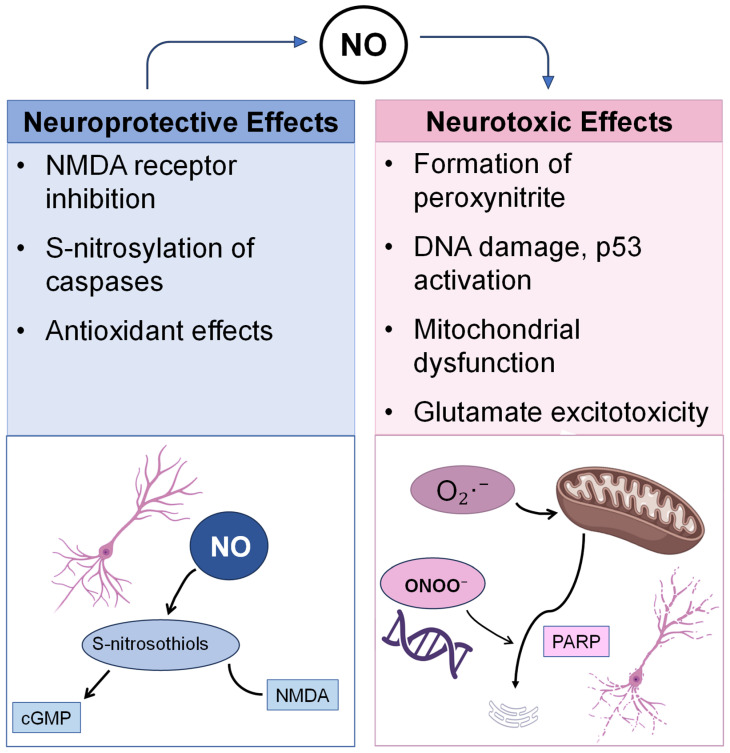
Overview of the dual (neuroprotective and neurotoxic) effects of nitric oxide (NO) in the central nervous system (CNS). Neuroprotective actions include inhibition of NMDA receptor activity via S-nitrosothiol formation, S-nitrosylation of caspases, antioxidant effects, and signaling through cyclic GMP (cGMP). Conversely, excessive NO contributes to neurotoxicity through formation of peroxynitrite (ONOO^−^), induction of DNA damage and p53 activation, mitochondrial dysfunction, PARP overactivation, and glutamate-mediated excitotoxicity. The balance between these antagonist actions governs neuronal survival or death. Nitric oxide (NO); N-methyl-D-aspartate (NMDA); cyclic GMP (cGMP); superoxide anion (O_2_·^−^); peroxynitrite anion (ONOO^−^); poly (ADP-ribose) polymerase (PARP).

Since its discovery [49], extensive research on NO has revealed its dual role as both a neuroprotective and neurotoxic molecule (Figure 1) [50,51]. Under conditions of sustained elevation of extracellular Ca^2+^, persistent NO production increases the risk of oxidative stress [52,53]. One major pathway of NO-induced neurotoxicity includes its involvement in ONOO^−^ synthesis, a harmful species that plays a central role in NO-mediated cell death [54,55]. ONOO^−^ induces single-strand DNA breaks, triggering activation of nuclear poly (ADP-ribose) polymerase (PARP). Excessive PARP activity transfers ADP-ribose from NAD^+^ to histones or PARP itself, depleting ATP and causing energy failure, ultimately leading to cell death [56,57]. Additionally, NO-induced DNA damage upregulates the tumor suppressor protein p53, promoting apoptosis [58,59].

Mitochondria are particularly vulnerable to the deleterious effects of NO because NO readily diffuses across the mitochondrial membrane where it can react with O_2_·^−^ that is continuously generated by the electron transport chain. ONOO^−^ inactivates key mitochondrial enzymes, such as succinate dehydrogenase, NADH dehydrogenase, and mitochondrial ATPase, irreversibly impairing cellular respiration [60]. It also inactivates Mn-superoxide dismutase (Mn-SOD), further increasing ONOO^−^ production [61], and oxidizes thiols and NADPH, inducing mitochondrial permeability transition pore (PTP) opening. This leads to Ca^2+^ efflux [62,63], loss of mitochondrial integrity, and cell death [64]. In neurons, ONOO^−^ nitrates tyrosine residues in neurofilaments, causing axonal damage, and inhibits glutamate transporters in glia, elevating extracellular glutamate. Excess glutamate overstimulates glutamate receptors, leading to Ca^2+^ influx and excitotoxicity, a mechanism implicated in various neurodegenerative disorders [65,66].

Conversely, NO can also mediate neuroprotection. It protects nNOS-containing neurons [67,68], and, when delivered via sodium nitroprusside, prevents NMDA-induced neurotoxicity in cultured cerebellar neurons [69]. NO attenuates NMDA-receptor-mediated damage by reacting with receptor complexes to form S-nitrosothiols, thereby inactivating NMDA receptors [34,70]. Additional neuroprotective mechanisms include: (I) S-nitrosylation of caspases and inhibition of PARP cleavage by caspases [71,72,73]; (II) antioxidant effects of NO and nitrosothiols [74,75]; (III) cyclic GMP-mediated effects, which can counter oxidative injury or trophic factor deprivation [76,77,78]; and (IV) induction of GSH synthesis [79]. However, the role of cyclic GMP remains controversial: some studies report no involvement in NO-mediated protection [80], while others suggest that it may contribute to NO-induced neuronal toxicity [81].

Taken together, these findings illustrate that NO acts as a double-edged sword in the nervous system; its balance between protective and damaging effects depends on concentration, cellular context, and redox environment, making it a key mediator in the interplay between oxidative stress and neurodegeneration (Figure 1).

### 3.1. Oxidative Stress and Nitrosative Stress and Their Role in Neurodegeneration

Oxidative stress, resulting from an imbalance between ROS production and antioxidant defenses, is a key driver of neurodegeneration, contributing to neuronal dysfunction, protein misfolding, and cell death. Misfolded and aggregated proteins, gliosis, and chronic neuroinflammation are major pathological outcomes. Neurodegenerative diseases also exhibit selective neuronal vulnerability [82,83]: in AD, neurons of the entorhinal cortex, hippocampal CA1, frontal cortex, and amygdala are most affected [67,84,85]; in PD, dopaminergic neurons of the *substantia nigra* are highly susceptible [86,87,88]; and in amyotrophic lateral sclerosis (ALS), degeneration primarily targets spinal, cortical, and brainstem motor neurons [89]. Subregional differences are also evident, such as the greater vulnerability of hippocampal CA1 versus CA3 neurons to oxidative stress, ischemia, seizures, and aging [90,91,92]. Neurons are especially susceptible to oxidative stress due to their high oxygen consumption, extensive mitochondrial activity, and limited regenerative capacity.

Under normal physiological conditions, approximately 1–2% of consumed oxygen is converted into ROS, but this percentage increases significantly with aging [93,94]. The oxidative burden results in the peroxidation of lipids, oxidation of proteins and nucleic acids, and generation of toxic byproducts such as aldehydes, ketones, and cholesterol oxides [95,96,97,98]. Notably, unsaturated lipids in neuronal membranes are particularly prone to oxidative modification, and lipid peroxidation is widely used as a sensitive biomarker of oxidative damage.

As previously mentioned, one key redox-regulated mechanism affected by oxidative stress is S-nitrosylation, a post-translational modification involving the covalent attachment of NO-related species to protein thiol groups. This modification can profoundly influence protein function and signaling. While physiological S-nitrosylation is essential for cellular homeostasis, pathological S-nitrosylation, especially via ONOO^−^ generated during aging, can lead to irreversible protein damage and membrane disruption. In the CNS, excessive or aberrant S-nitrosylation has been implicated in neurodegenerative mechanisms, promoting protein misfolding and aggregation in disorders such as AD and PD [99].

Moreover, oxidative stress impairs the proteasomal degradation system, the primary mechanism for removing oxidized or misfolded proteins. Accumulation of oxidatively modified proteins, particularly those that are cross-linked, can inhibit proteasomal activity, leading to further protein aggregation and cellular toxicity [100]. This dysfunction creates a self-perpetuating loop of proteostasis failure.

In parallel, oxidative stress activates glial cells, microglia and astrocytes, promoting a neuroinflammatory milieu characterized by the release of additional ROS, NO and proinflammatory cytokines.

This feedback loop exacerbates neuronal damage and inflammation. In neurodegenerative conditions such as AD, PD and ALS, these combined effects drive pathological cascades including Aβ deposition, tau hyperphosphorylation, alpha-synuclein aggregation, and progressive neuronal loss, ultimately resulting in cognitive and motor decline.

### 3.2. Nitric Oxide and Alzheimer’s Disease

AD is the most prevalent form of dementia, characterized by progressive neurodegeneration, synaptic and neuronal loss, cognitive decline, and, ultimately, death [101]. The neuropathological hallmarks of AD include extracellular -Aβ plaques and intracellular neurofibrillary tangles composed of hyperphosphorylated tau protein [102,103]. Dysregulation of NO signaling has been implicated as a contributing risk factor in AD, particularly in the context of aging [77]. NO is essential for proper neuronal communication and synaptic plasticity, but its imbalance in AD contributes to pathological processes.

In AD, NO dysregulation enhances Aβ plaque deposition and exacerbates synaptic dysfunction and chronic neuroinflammation. This process is mediated by the excessive generation of ROS, notably ONOO^−^, which accelerates oxidative damage and disrupts synaptic signaling [104]. Aβ itself can further induce ROS production [105], establishing a self-amplifying cycle of oxidative stress and Aβ accumulation. ROS also promote amyloidogenic processing of amyloid precursor protein (APP) by upregulating BACE1 via activation of stress-activated protein kinases such as c-Jun N-terminal kinase (JNK) and p38 mitogen-activated protein kinase (p38-MAPK) [106,107].

Additionally, Aβ oligomers stimulate the overexpression of iNOS in astrocytes, increasing NO production [108]. Aβ also impairs glutamate reuptake, leading to pathological activation of NMDA receptors, calcium influx, and subsequent nNOS activation, contributing to NO overload [109,110]. NO plays a dual role in AD pathogenesis: (I) it modulates the release of proinflammatory cytokines and interacts with ROS to form RNS, which are associated with neuroinflammation and oxidative stress-mediated neurodegeneration [111]; (II) it mediates the S-nitrosylation process, involved in protein function alteration. Aberrant S-nitrosylation has been implicated in various neurodegenerative disorders, including AD [30,99,112]. Notably, dysregulated S-nitrosylation pathways are increasingly recognized as potential drivers of AD pathophysiology [30].

For instance, Cheng et al. reported that S-nitrosylation of the Cdk5 activator p39 is elevated in Aβ-treated neurons and APP/PS1 transgenic mice, contributing to synaptic deficits and dendritic spine loss, thereby implicating NO-mediated p39 degradation in AD-related synaptic dysfunction [113]. Furthermore, Yang et al. demonstrated the presence of aberrant S-nitrosylation in human AD brain samples from both sexes, providing direct evidence of this mechanism in AD pathology [114]. Recent findings by Suloh et al. suggest that altered S-nitrosylation during early AD stages affects glutamatergic/GABAergic neurotransmission and mTOR signaling in two mouse models of AD and tauopathy, highlighting its potential as a biomarker and therapeutic target in early-stage AD [115]. Importantly, Qu et al. discovered that S-nitrosylation of deubiquitinase ubiquitin carboxy-terminal hydrolase isoenzyme-L1 led to synaptic damage in AD, highlighting NO-mediated S-nitrosylation of proteins as instrumental in AD progression [116].

Another hallmark of AD closely linked to NO signaling is oxidative and nitrosative stress, which leads to the accumulation of free radicals in the brain, a feature measurable as a clinical marker in AD brain [117]. Oxidative stress enhances phosphorylation of Aβ and tau, and damages nucleic acids, while nitrosative stress results from the interplay of ROS and RNS, generating reactive intermediates, such as 3-nitrotyrosine [118]. These species induce neuronal damage via tyrosine nitration, mitochondrial dysfunction, and membrane disruption. Elevated levels of 3-nitrotyrosine, up to sixfold higher, have been consistently observed in AD brain tissue and cerebrospinal fluid, underlining the contribution of nitrosative stress to neurodegeneration [119]. Shared molecular features of neurodegenerative diseases include oxidative/nitrosative stress, inflammation, and protein aggregation. Aβ accumulation initiates inflammatory responses involving microglia and astrocytes [120,121], which phagocytose Aβ aggregates and secrete proinflammatory cytokines.

These processes activate the complement system and promote ROS generation [122]. Activated microglia release neurotoxic NO in response to Aβ deposition [123], while Aβ disrupts mitochondrial membranes, impairing energy metabolism and contributing to synaptic failure [124]. Chronic inflammation perpetuates high iNOS expression, further increasing NO levels and accelerating Aβ deposition [125]. Excess NO also alters NMDA receptor expression and function, enhancing excitotoxicity and promoting neuronal death [126].

**Figure 2:**
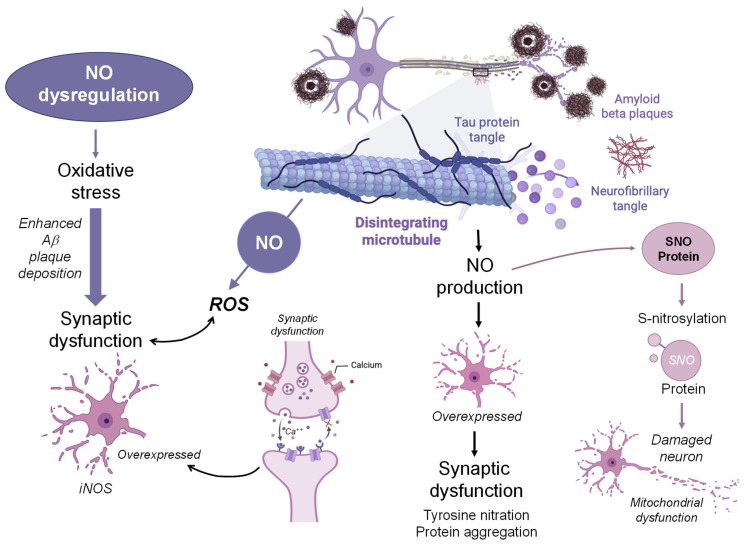
The contribution of nitric oxide (NO)-induced neurotoxicity in Alzheimer’s disease (AD) pathology. Increased NO production, caused by inducible NOS (iNOS) and Ca^2+^-dependent neuronal activation, boosts oxidative stress and reactive oxygen species (ROS) generation. This fosters amyloid-β (Aβ) plaque deposition, tau hyperphosphorylation and aggregation into neurofibrillary tangles, mitochondrial dysfunction, and synaptic impairment. NO-mediated protein S-nitrosylation and tyrosine nitration further alter protein function and promote aggregation. These interconnected processes form a self-amplifying cycle that drives neuronal damage and progression of AD pathology. Amyloid-beta (Aβ); inducible NO synthase (iNOS); reactive oxygen species (ROS); S-nitrosothiol (SNO).

Importantly, NO is essential for memory consolidation via its role in long-term potentiation (LTP) in the hippocampus and cortex. Acting as a retrograde messenger downstream of NMDA receptor activation, NO facilitates synaptic strengthening crucial for learning and memory [127,128]. The involvement of NO in AD remains controversial, largely due to its dual role in the central nervous system (Figure 2).

On one hand, NO exhibits neuroprotective properties through activation of the cGMP pathway [129,130]. This signaling enhances cerebral blood flow, limits oxidative stress, and inhibits NMDA receptor-mediated calcium influx at glutamatergic synapses. On the other hand, under pathological conditions, NO is responsible for ONOO^−^ production, which is involved in lipid peroxidation, calcium homeostasis impairment, and S-nitrosylation, which are all molecular events linked to AD pathology [131,132,133,134]. Prolonged upregulation of constitutive NOS can cause the NOS homodimer to uncouple, favoring ONOO^−^ production over cGMP-mediated signaling [135]. Measuring NO and ONOO^−^ in situ remains challenging due to their short half-lives (<3 s and <1 s, respectively), complicating efforts to distinguish protective from toxic NO signaling [136]. Nevertheless, advances in nanotechnology-based detection have clarified that NO’s cytotoxicity in AD primarily emerges after its conversion to ONOO^−^ [137].

Altogether, these observations highlight that NO itself is not inherently neurotoxic in AD, but its pathological conversion to ONOO^−^ under oxidative stress conditions is a key driver of neurodegeneration.

### 3.3. Nitric Oxide and Tauopathy

Tauopathies are a group of neurodegenerative disorders defined by the pathological accumulation, hyperphosphorylation, and aggregation of the tau protein in neurons and glia. Tau is a microtubule-associated protein (MAP) encoded by the MAPT gene on chromosome 17q21.31 [138], and its primary role is to promote microtubule (MT) assembly and stabilize axonal MTs [139,140]. Tau is a phosphoprotein with multiple phosphorylation sites on serine and threonine residues [141], particularly in regions flanking its MT-binding domains. Hyperphosphorylation of tau impairs its ability to bind microtubules [142], facilitating its aggregation into neurofibrillary tangles (NFTs), a pathological hallmark of AD and other tauopathies [143]. Oxidative stress, often resulting from the accumulation of ROS, is a common feature of tauopathies and has been shown to exacerbate tau pathology [144,145]. ROS can oxidize lipids, proteins, and nucleic acids, contributing to cellular damage. However, it remains unclear whether oxidative stress is a primary trigger or a downstream consequence of tau-induced neurodegeneration. Multiple models of tauopathies have suggested that overexpression of mutant human tau increases ROS levels and sensitizes neurons to oxidative insults, such as H_2_O_2_.

In N2a neuroblastoma cells, overexpression of wild-type tau led to enhanced vulnerability to oxidative stress, attributed to impaired peroxisomal transport along destabilized microtubules [146]. Similarly, a different work showed that oxidative stress in neuroblastoma cells promotes tau hyperphosphorylation via microtubule affinity-regulating kinases (MARKs), responsible for the regulation of tau–microtubule binding and thereby playing a crucial role in neuronal survival [147]. Proteomic analysis of transgenic tau P301L mice, a model of FTD with parkinsonism linked to chromosome 17 (FTDP-17), revealed downregulation of proteins involved in mitochondrial oxidative phosphorylation (particularly complex V subunits) and antioxidant defense systems, including peroxiredoxins 5/6, GSH S-transferase, and GPx. These mice exhibited increased ROS levels, lipid peroxidation, elevated antioxidant enzyme activity, and mitochondrial dysfunction, with ROS accumulation intensifying between 12 and 24 months of age in parallel with tau pathology progression [148]. Conversely, other studies have reported that oxidative stress may precede tau aggregation, including in early-stage AD and other tauopathies [149,150]. In vitro, ROS and related molecules like 4-hydroxynonenal (4-HNE) have been shown to enhance tau phosphorylation [151] and promote aggregation of phosphorylated tau [126,152,153].

NO contributes to tau pathology through both direct and indirect mechanisms. One of the primary direct effects is the S-nitrosylation of tau and tau-regulatory enzymes, such as kinases and phosphatases, which alters tau’s conformation, impairs microtubule binding, and enhances aggregation [154]. In a mouse model of tauopathy (P301S mice), increased levels of PSNOs, including kinases and transcription factors, were detected in the cortex and hippocampus. Gene ontology analysis indicated that these PSNOs are associated with neuronal and synaptic functions, such as nervous system development, neurogenesis, and vesicle-mediated transport.

Furthermore, MetaCore (Clarivate Analytics), a web-based bioinformatics software analysis identified both primary and secondary interactions between tau and these PSNOs [112]. Indirectly, NO and NO-derived RNS may promote tau hyperphosphorylation by (I) inhibiting phosphatases like PP2A through S-nitrosylation or oxidative mechanisms, and (II) activating redox-sensitive kinases such as GSK-3β and CDK5 [155,156,157].

**Figure 3:**
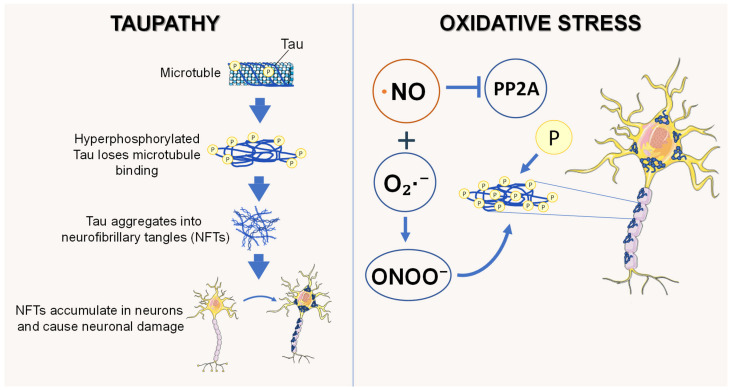
The mechanism of nitric oxide (NO)-induced neurotoxicity in tauopathy. NO reacts with superoxide (O_2_·^−^) to generate peroxynitrite (ONOO^−^), promoting oxidative stress and inhibiting protein phosphatase 2A (PP2A), a key regulator of tau dephosphorylation. This imbalance causes tau hyperphosphorylation, loss of microtubule binding, and aggregation into neurofibrillary tangles (NFTs). Accumulation of NFTs disrupts neuronal structure and function, ultimately steering to neuronal damage and degeneration. Neurofibrillary tangles (NFTs); NO radical (·NO); superoxide anion (O_2_·^−^); peroxynitrite anion (ONOO^−^); protein phosphatase 2A (PP2A).

Additionally, ONOO^−^ produced by NO is capable of nitrating tyrosine residues in tau, which further destabilizes its structure and promotes oligomer formation [158,159,160] (Figure 3).

Emerging evidence also indicates that TDP-43, another key pathological protein implicated in frontotemporal lobar degeneration (FTLD) and ALS, shares overlapping stress-related pathways with tau. Both proteins are susceptible to oxidative and nitrosative post-translational modifications, which can promote their misfolding, aggregation, and cytoplasmic mislocalization. These findings highlight the complex interplay between oxidative and nitrosative stress and tau pathology, suggesting that NO and its derivatives may act as both modulators and amplifiers of tau-driven neurodegeneration.

### 3.4. Nitric Oxide and Frontotemporal Lobar Degeneration

FTLD is a heterogeneous neurodegenerative disorder with a strong genetic basis, as up to 40% of patients report an affected family member [161]. In 1994, an autosomal dominant form of frontotemporal dementia (FTD) with parkinsonism was linked to chromosome 17q21.2 [162], and subsequent studies identified additional familial cases associated with this locus, termed “frontotemporal lobar degeneration and parkinsonism linked to chromosome 17” (FTDP-17). Mutations in the MAPT gene, encoding microtubule-associated protein tau, were found to cause the disease in these families [163,164], with 44 distinct variants reported across 132 families. Up to 50% of FTDP cases are familial, most commonly with autosomal dominant inheritance, and the main genetic causes include mutations in MAPT, GRN (progranulin), and C9orf72 (chromosome 9 open reading frame 72).

The C9orf72 repeat expansion, found in both FTLD and ALS, is strongly linked to the accumulation of the RNA-binding protein TAR DNA-binding protein 43 (TDP-43). In ~95% of ALS cases and 50% of FTLD cases, cytoplasmic aggregates of TDP-43 are a pathological hallmark [165,166,167]. Normally, TDP-43 is a predominantly nuclear protein involved in transcription, splicing, microRNA biogenesis, and RNA transport [168,169,170]. In FTD/ALS, however, it mislocalizes to the cytoplasm, forming aggregates and depleting its nuclear pool [171,172]. Although the triggers of this mislocalization remain incompletely understood, the discovery of the C9orf72 repeat expansion as the most common genetic cause of FTD and ALS (c9FTD/ALS) has advanced our understanding of the shared molecular pathology [173,174].

Two major risk factors in neurodegenerative disease, aging and environmental toxins, are known to increase ROS and RNS, including NO [19,175]. Consequently, NO has been implicated in ALS/FTD pathogenesis [19,176,177]. Notably, environmentally induced nitrosative stress promotes TDP-43 aggregation and neurotoxicity via cell-to-cell spread. NO-related species S-nitrosylate TDP-43 at Cys173 or Cys175, enable disulfide bond-mediated aggregation, and impair its function. Pathological SNO–TDP-43 has been found to be present in postmortem FTD/ALS brains and in cell-based models, including human-induced pluripotent stem cell-derived neurons. Aggregated TDP-43 further amplifies nitrosative stress in a feed-forward cycle, promoting additional aggregation, in vivo spread, RNA-binding loss, and dysregulation of SNMT1 and pCREB, thereby contributing to neuronal injury [178]. Moreover, transcriptome analysis identified Nitric Oxide Synthase 1 Adaptor Protein (NOS1AP) mRNA as a direct TDP-43 target, with its downregulation in primary mouse cortical neurons disrupting NMDA receptor signaling [179].

Collectively, these findings suggest that NO-driven nitrosative stress not only promotes pathological TDP-43 aggregation and spread but also disrupts neuronal signaling pathways, positioning redox imbalance as a central mechanism linking oxidative stress to neurodegeneration in ALS/FTD.

### 3.5. Nitric Oxide and Amyotrophic Lateral Sclerosis (ALS)

ALS is a fatal neurodegenerative disorder characterized by degeneration of upper and lower motor neurons, leading to progressive loss of voluntary muscle control. More than 50 disease-associated genes have been identified, with the most frequent mutations occurring in superoxide dismutase 1 (SOD1), C9orf72, TAR DNA-binding protein (TARDBP) (TDP-43), and fused in sarcoma (FUS) [180]. Sporadic ALS (sALS) accounts for ~90–95% of cases, while familial ALS (fALS), often with an earlier onset, represents 5–10% [181,182]. Although the precise pathogenic mechanisms remain unclear, fALS and sALS likely share converging pathways, including glutamate excitotoxicity, RNA metabolism defects, impaired axonal transport, protein misfolding/aggregation, endoplasmic reticulum (ER) stress, disrupted protein trafficking, oxidative stress, inflammation, and mitochondrial dysfunction [44,183,184].

Given the previously described link between NO and TDP-43 pathology, this section focuses on the interplay between NO and mutant SOD1. SOD1 encodes a Cu/Zn metalloprotein that converts O_2_·^−^ into oxygen and H_2_O_2_, functioning mainly in the cytosol but also present in the nucleus, peroxisomes, and mitochondria. SOD1 is central to antioxidant defense [185] and also regulates cellular respiration and energy metabolism [186]. However, SOD1 can act as a peroxidase, either catalyzing the reverse of its dismutase reaction or using H_2_O_2_ as a substrate in a Fenton-type reaction to generate hydroxyl radicals (·OH) [187,188]. Mutant SOD1 (mutSOD1) can further enhance oxidative damage via zinc dissociation from the enzyme [189] or by exposing reactive copper at its active site, promoting aberrant O_2_·^−^ production, involved in ONOO^−^ synthesis [164,165,190]. Extensive in vitro and in vivo studies support the concept that copper in SOD1 can interact with NO and ONOO^−^ to initiate motor neuron death [191,192].

**Figure 4:**
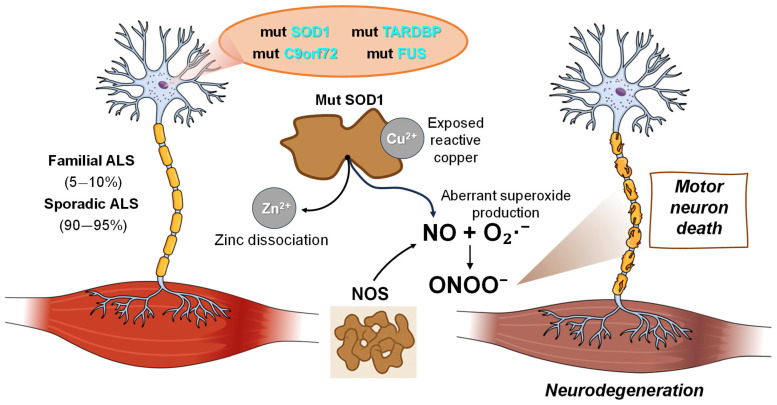
Crosstalk between nitric oxide (NO) and mutant superoxide dismutase 1 (mutSOD1) in amyotrophic lateral sclerosis (ALS) pathogenesis. Mutations in SOD1 and other ALS-associated genes (e.g., C9orf72, TARDBP, FUS) induce zinc dissociation and exposure of reactive copper, enhancing aberrant redox activity and aberrant superoxide production. NO reacts with superoxide to form peroxynitrite (ONOO^−^), driving oxidative damage and motor neuron degeneration. These processes contribute to both familial and sporadic ALS, culminating in progressive motor neuron death and consequently neurodegeneration. Superoxide dismutase 1 (SOD1); chromosome 9 open reading frame 72 (C9orf72); TAR DNA-binding protein (TARDBP); fused in sarcoma (RNA-binding protein) (FUS); superoxide anion (O_2_·^−^); peroxynitrite anion (ONOO^−^).

On the neuroprotective side, motor neurons expressing eNOS when exposed to brain-derived neurotrophic factor (BDNF) survive via NO binding to the heme group of sGC, stimulating cGMP production [193]. Inhibition of NO synthesis under these conditions decreases survival, whereas exposure to low-steady-state NO (<100 nM) or cGMP analogs restores protection (Figure 4).

Conversely, in trophic factor-deprived motor neurons, NO production increases via nNOS upregulation, accompanied by O_2_·^−^ co-generation. This drives endogenous ONOO^−^ formation, increased nitrotyrosine immunoreactivity, and apoptosis [193]. NO-dependent cGMP activation can block apoptosis by suppressing nNOS expression through sequestration of intracellular calcium [194], while NO inhibition reduces nitrotyrosine accumulation and cell death [195]. Scavenging O_2_·^−^ is similarly protective, indicating that NO alone is insufficient to trigger apoptosis [193]. Fas signaling also contributes to NO-mediated motor neuron death [196]. Fas activation engages two parallel pathways: (I) nNOS induction via FADD, and (II) caspase-8-driven mitochondrial apoptosis cascades [197,198,199].

In mutSOD1-expressing neurons with sufficient trophic support, low-level NO activates Fas/FasL signaling, upregulates nNOS, and drives further NO production, creating a positive feedback loop that promotes cell death [200]. This loop may underlie the slow, progressive motor neuron loss in ALS. nNOS upregulation precedes iNOS induction in both ALS patients and presymptomatic animal models [201,202], and is observed after axonal injury alongside increased nitrotyrosine [203,204]. NOS inhibition protects motor neurons from ventral root avulsion, while SOD1 deficiency increases vulnerability [203,205].

Overall, NO/Fas-mediated signaling is a critical driver of apoptosis in cultured and in vivo motor neurons. While NO alone does not initiate apoptosis, its reaction with O_2_·^−^, producing ONOO^−^, can both (I) inhibit cGMP-mediated neuroprotection and (II) promote oxidative stress, ultimately leading to motor neuron degeneration in ALS.

### 3.6. Nitric Oxide and Parkinson’s Disease (PD)

PD is one of the most common age-related neurodegenerative disorders, characterized by the progressive loss of dopaminergic neurons in the *substantia nigra pars compacta* (SNpc), leading to dopamine depletion in the striatum. Although the precise etiology and pathogenic mechanisms remain unclear, mitochondrial impairment, particularly a marked reduction in complex I activity of the mitochondrial respiratory chain, has been reported to be involved in PD pathogenesis [206,207].

Impaired complex I function reduces ATP synthesis, disrupting neuronal energy metabolism. Given neurons’ high energy demands and limited glycolytic capacity, such ATP depletion can cause partial depolarization, persistent activation of NMDA glutamate receptors, and sustained Ca^2+^ influx, triggering secondary excitotoxicity [208,209]. In this context, a recent work showed that the LRRK2 G2019S mutation in PD further exacerbates neuronal vulnerability by inducing ER stress, which is associated with Ca^2+^ dysregulation and mitochondrial impairment [210].

Mitochondrial dysfunction also increases ROS generation, including O_2_·^−^, NO, H_2_O_2_, and ONOO^−^ [211]. Accordingly, LRRK2-driven ER stress causes mitochondrial dysfunction and ROS generation, activating oxidative stress pathways in PD. Notably, ER stress-associated ROS may synergize with NO, enhancing the formation of reactive nitrogen species such as ONOO^−^ [210].

In PD, excessive ROS and NO drive ONOO^−^ formation, leading to oxidative and nitrative modifications of proteins, lipids, and nucleic acids, thereby impairing their structure and function [17,212]. Evidence for nitroxidative stress in PD includes elevated levels of malondialdehyde (MDA), a lipid peroxidation product [213], and 8-hydroxy-2′-deoxyguanosine, a marker of oxidative DNA damage [214], in the *substantia nigra* of PD patients compared with other parkinsonian brain regions and controls. Increased protein carbonyl content, an indicator of oxidative stress, and protein nitration are also observed, notably involving α-synuclein [215,216].

The selective vulnerability of dopaminergic neurons suggests that dopamine and its metabolites play a direct role in neurodegeneration. Cytosolic dopamine, arising from synaptic reuptake or vesicular leakage, induces toxicity through multiple mechanisms: (I) inhibition of the mitochondrial respiratory chain [217]; (II) ROS, quinone, and semiquinone formation via monoamine oxidase (MAO) metabolism or auto-oxidation; (III) depletion of antioxidants such as GSH [218].

The role of NO in PD pathogenesis is supported by several findings: (I) overexpression of nNOS in PD brains [219]; (II) activation of microglia expressing iNOS before dopaminergic neuron death [220]; (III) neuroprotection by nNOS and iNOS inhibition in animal PD models [221]; (IV) elevated 3-nitrotyrosine levels, a marker of ONOO^−^, in PD patients and models [215].

A bidirectional interaction between abnormal dopamine metabolism and excessive NO production amplifies neurotoxicity. This includes:

1. Glutamate-driven potentiation of both NO production and dopamine toxicity [222].
2. NO–dopamine interactions such as (i) S-nitrosylation of tyrosine hydroxylase (TH), increasing enzyme activity, (ii) enhanced dopamine release and reuptake inhibition, and (iii) dopamine oxidation directly or via ONOO^−^.
3. Dopamine-induced stimulation of mitochondrial NO production [223].
4. Dopamine enhancement of NO-induced mitochondrial dysfunction in PC-12 cells [224].

**Figure 5:**
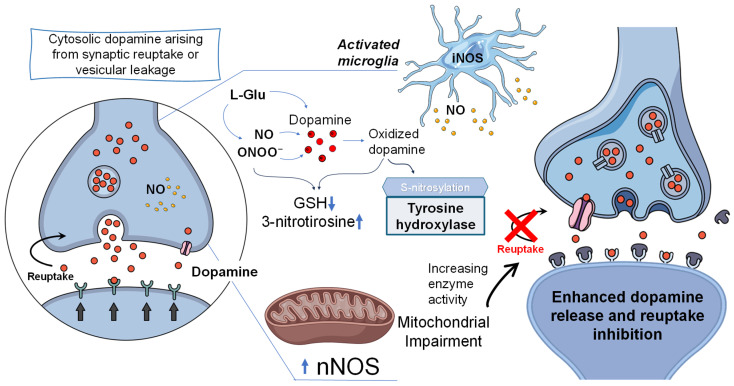
Schematic diagram of NO–dopamine–mitochondria interaction in Parkinson’s disease (PD). Cytosolic dopamine derived from synaptic reuptake or vesicular leakage undergoes oxidation, generating reactive species and contributing to oxidative stress. NO, produced by neuronal NOS (nNOS) and inducible NOS (iNOS) in activated microglia, reacts with superoxide to form peroxynitrite (ONOO^−^), causing protein nitration (e.g., 3-nitrotyrosine formation), glutathione (GSH) depletion, and mitochondrial dysfunction. Moreover, NO-mediated S-nitrosylation of tyrosine hydroxylase enhances dopamine synthesis, while increased dopamine release and impaired reuptake elevate cytosolic dopamine levels, further amplifying oxidative stress. This self-reinforcing cycle contributes to synaptic dysfunction and progressive dopaminergic neuron degeneration in PD. Glutamate (L-Glu); peroxynitrite anion (ONOO^−^); glutathione (GSH); inducible NO synthase (iNOS); neural NO synthase (nNOS).

The interplay between NO and dopamine metabolism is particularly relevant within mitochondria. The dopamine metabolite 3,4-dihydroxyphenylacetic acid (DOPAC) is produced in the mitochondrial matrix through sequential MAO and aldehyde dehydrogenase (ALDH) activity, with the latter expressed in a subset of dopaminergic neurons [225]. Mitochondrial NOS (mtNOS), a proposed mitochondrial-associated source of NO whose existence and molecular identity remain debated, has been reported in proximity to complex I, where it may produce NO, which in turn oxidizes DOPAC to form potentially toxic species such as nitroxyl anion (NO^−^), o-semiquinone radicals, and o-quinones [226] (Figure 5).

In summary, excessive NO production and aberrant dopamine metabolism converge on a common toxic cascade involving mitochondrial dysfunction, nitroxidative stress, and GSH depletion. This synergy likely contributes to the progressive neurodegeneration observed in PD.

As outlined in the previous sections, AD, ALS, tauopathies, and PD exhibit overlapping mechanisms of NO-mediated oxidative and nitrosative stress that drive neurodegenerative processes. The common and disease-specific pathways underlying these mechanisms are summarized in Table 1.

### 3.7. Gender Influence on Nitrergic Signaling in Neurodegenerative Disease

It is well-established that gender significantly influences oxidative stress responses [227,228,229]. Specifically, sex steroid hormones such as estrogen and testosterone have been shown to contribute to gender-related differences observed in neurodegenerative disorders [230]. In contrast, the impact of gender on NO-induced oxidative and nitrosative stress remains poorly characterized, mainly because the majority of experimental studies have been performed in male animals. Nevertheless, accumulating evidence indicates that both sex hormones and sex-linked genetic factors differentially regulate NO production as well as oxidative and nitrosative stress pathways across distinct brain regions [231,232,233], underlying the possible role of NO as a gender-difference mediator during development [234]. For instance, it was described that estradiol positively regulates hippocampal NO production via estrogen receptor-mediated nNOS expression, whereas stress increases glucocorticoid-dependent NO production in males but suppresses it in females due to reduced estrogen levels [235]. Gender-dependent differences in nNOS expression and NO-related responses have been observed across several brain regions and pathological conditions. Females generally exhibit greater resistance to oxidative and nitrosative stress, while males show higher oxidant production, partly attributed to testosterone-related effects [233,236,237,238]. Proteomic and transcriptomic analyses further reveal gender-specific molecular signatures in ischemia, microglial function, and hippocampal development, highlighting distinct pathways related to synaptic signaling, immune responses, metabolism, and protein homeostasis [239,240,241]. Notably, the most important gender-related difference in oxidative stress, mainly NO production and nitrosative stress, is reported in AD and PD [242]. Concerning PD, augmented oxidative stress mediated by NADPH-oxidase was reported in male rat dopaminergic neurons in the *substantia nigra* as compared to female rats [243]. Additionally, testosterone has been shown to be an important mediator of oxidative stress in dopaminergic neurons [244,245], exacerbating motor impairments [246]. Furthermore, testosterone may contribute to the increased incidence of PD observed in post-menopausal women compared to pre-menopausal women [247], particularly by shifting toward a relatively more androgenic and less estrogenic hormonal milieu after menopause [248,249], highlighting the influence of hormonal imbalance on NO production and redox homeostasis, triggering nitrosative stress and contributing to dopaminergic vulnerability. Regarding AD, whose incidence is significantly higher in women [250], several publications have described the neuroprotective role of estrogen against oxidative stress induced by Aβ and APP [242,251,252,253].

The strong influence of gender on nitrosative stress in AD has been described by Yang et al. [114], who identified an extensive remodeling of the brain S-nitrosoproteome, with a remarkable female-specific increase in S-nitrosylation of complement component C3, inducing excessive microglia-mediated synaptic phagocytosis in female AD brains. Importantly, estradiol suppressed SNO-C3 formation by limiting iNOS-derived NO production, underlining that estrogen loss induces aberrant C3-nitrosylation and suggesting molecular crosstalk between NO dysregulation, complement activation, and female vulnerability in AD [114]. Finally, a recent study proposed the concept of “mutational mimicry,” whereby aberrant protein SNO phenocopies the functional effects of rare genetic mutations in neurodegenerative diseases [254]. Specifically, through the integration of human S-nitrosoproteomics with GWAS data, a significant overlap was detected between abnormally S-nitrosylated proteins and genetic risk factors in neurological disorders (AD, PD, and ALS). Pathological SNO impairs mitochondrial function, proteostasis, autophagy, and synaptic integrity, placing nitrosative stress as a mechanistic link between environmental triggers and inherited susceptibility. Together, these findings underscore an important gender-dependent regulation of NO signaling, oxidative stress responses, and brain molecular networks, with important implications for disease vulnerability. Incorporating gender-specific NO biology is therefore essential for developing precision diagnostics and personalized therapies in neurodegenerative disorders.

## 4. Antioxidants in Therapeutic Approaches

Growing evidence highlights antioxidants as a promising therapeutic avenue for neurodegenerative disorders, particularly those involving dysregulation of the NO/NOS system. Since excessive NO production contributes to neuronal injury, oxidative stress, and disease progression, while low physiological levels remain essential for normal neuronal signaling, the therapeutic challenge lies in achieving a controlled reduction in NO synthesis rather than complete inhibition.

This fine-tuning may help preserve neuronal function while limiting neurodegeneration. A variety of synthetic compounds, such as amino acid amidine derivatives, urea, thiourea, other urea derivatives, aromatic amidines, and cyclic amidines, have been investigated for their NOS-modulating potential [255,256,257,258]. In parallel, natural and nutraceutical compounds with antioxidant properties, including polyphenols, flavonoids, and plant-derived bioactives, have shown protective effects by scavenging ROS, reducing ONOO^−^ formation, and restoring redox homeostasis [259,260].

Among several natural compounds investigated for counteracting oxidative stress in AD, carnosine, a natural β-alanyl-L-histidine dipeptide, has emerged as one of the most promising agents for mitigating Aβ-induced oxidative stress [261,262]. In particular, the modulation of NO activity exerted by carnosine has been evaluated in different previous studies, in which the ability of the dipeptide to affect NO bioavailability and to regulate the iNOS enzyme was highlighted [261,262,263,264,265]. A study on NO metabolism demonstrated that carnosine directly interacts with NO through its β-alanine and histidine moieties, forming stable adducts and thereby accelerating the conversion of NO into NO_2_^−^, a minimally toxic NO end product. By favoring this reaction, carnosine was able to limit nitrosative stress without inhibiting iNOS activity. This chemical “buffering” of NO supports the anti-inflammatory and cytoprotective properties of carnosine under conditions of excessive NO production [265].

Building on these findings, carnosine was shown to dampen inflammation-induced increases in NO levels in macrophages, an effect correlated with a faster removal of NO from the cell environment, reducing the possibility of RNS production [260,266]. Beyond antioxidant activity, carnosine has been reported to exert marked neuroprotective effects by reducing microglial cell death caused by Aβ oligomers and promoting a shift toward an anti-inflammatory phenotype, characterized by decreased proinflammatory cytokine release and enhanced anti-inflammatory signaling. In particular, the direct modulation of NO synthesis pathways allowed a decrease in both NO and O_2_ ^−^ intracellular levels, along with reduced expression of iNOS and NADPH-oxidase. In this regard, these protective actions are further mediated by the induction of TGF-β1 signaling, ultimately preserving neuronal integrity and preventing amyloid-β-induced neurodegeneration [261].

More recently, these findings have been extended to primary mixed glial cultures, confirming carnosine’s role as a suppressor of Aβ-induced increase in intracellular NO, consistent with its ability to modulate iNOS-dependent nitrosative stress and glial inflammatory activation in AD [262]. Together, these results highlight the therapeutic potential of carnosine as a neuroprotective strategy to counteract Aβ-driven oxidative stress and neuroinflammation, two key pathological processes in AD, with possible relevance to neurodegenerative disorders more broadly [267].

Since neurodegenerative disorders often involve multiple NOS isoforms, iNOS, eNOS, and nNOS, less isoform-specific agents such as N5-(1-iminoethyl)-L-ornithine or 4-Methylaminopyridine may yield broader neuroprotective benefits [256,268,269,270,271].

Importantly, antioxidants could also indirectly support NO/NOS balance by preserving blood–brain barrier (BBB) integrity, enhancing mitochondrial function, and replenishing endogenous antioxidant defenses such as GSH.

These effects may limit the vicious cycle of oxidative and nitrosative stress that drives neuronal death in AD, tauopathies, FTD, ALS and PD. Despite encouraging preclinical results, translation to clinical practice has been slow due to the scarcity of well-designed human trials and the absence of NOS inhibitors or antioxidant-based NOS modulators approved for clinical use [256]. Ongoing trials exploring compounds that target oxidative and nitrosative stress through complementary mechanisms [272,273] may provide crucial breakthroughs (Table 2).

Given the complex interplay between NO/NOS signaling, redox imbalance, and neuronal survival, antioxidant strategies (especially those capable of crossing the BBB and modulating both mitochondrial and cytosolic sources of ROS/RNS) represent a promising and underexploited frontier in the treatment of neurodegenerative diseases. Of note, the heterogeneous contribution of NO-induced oxidative and nitrosative stress across disease stages and patient subgroups underlines the need for personalized antioxidant approaches. In this context, the continued integration of mechanistic insights from basic research with rigorous clinical evaluation will be essential to identify responsive patient populations and to fully realize the therapeutic potential of redox-targeted interventions.

## 5. Emerging Technologies and Therapeutic Strategies Targeting NO Signaling in Neurodegeneration

### 5.1. Microfluidic-Based Profiling of NO and Nitrosative Stress

Accurate quantification of NO and nitrosative stress species is essential to outline their dual neuroprotective and neurotoxic roles in neurodegeneration. Standard methodologies include fluorescent probe-based imaging, electrochemical detection, measurement of nitrite and nitrate by the Griess reaction or LC-MS/MS, and assessment of 3-nitrotyrosine and S-nitrosylated proteins through immunochemical or proteomic approaches [280,281,282,283,284]. However, the inherent challenges associated with NO analysis, including its short half-life and pronounced cell-to-cell heterogeneity in nitrosative stress, limit the resolution of conventional bulk assays. The integration of these analytical strategies into microfluidic platforms, defined as microscale fluid-based analytical devices [285,286], enhances sensitivity, reduces sample volume, and enables real-time and single-cell measurements. By resolving dynamic redox fluctuations and cellular heterogeneity, microfluidic technologies provide a powerful framework for precision NO profiling and personalized redox-based stratification in neurodegenerative diseases. To address the issue of NO’s short half-life, researchers fixed intracellular NO by using the well-established NO-specific fluorogenic probe 4-amino-5-methylamino-2′,7′-difluorofluoescein diacetate (DAF-FMDA) [262,285,286,287,288,289,290]. Although DAF-FMDA has been used previously to measure intracellular NO through fluorescence imaging, researchers were able to perform a more rigorous quantitation by incorporating an internal standard dye, 6-carboxyfluorescein diacetate (6-CFDA), and separating the two fluorophores via a micro-electrophoretic separation. This allowed for signal correction for natural variances in dye uptake and dye conversion by intracellular esterases. This method has been applied to the measurement of intracellular NO in bulk-cell lysate samples as well as in individual cells, or single-cell analysis (SCA) [288].

The SCA study revealed information about the shape of the population distribution for intracellular NO levels which may enable researchers to better understand natural cell-to-cell variation in NO production. In addition to measuring intracellular NO, microfluidics coupled with electrochemical detection has been employed to quantify nitrosative stress through the measurement of nitrite (a direct byproduct of NO production) or nitrated amino acid residues, like 3-nitro-l-tyrosine, that result from prolonged nitrosative stress [291,292,293]. Overall, microfluidic-based analysis represents a promising avenue toward the development of measurement tools with the level of precision that is required to better understand the minimal differences among cells/individuals with the final aim of developing targeted therapy.

### 5.2. Targeting NO Signaling for Personalized Therapeutic Strategies

NO represents a suitable candidate for personalized therapeutic approaches in neurodegenerative diseases because its fine regulation and downstream effects can result in different outcomes among individuals. Differences in NO availability, NOS isoform expression, redox balance, and cellular susceptibility have been reported across patients and disease conditions, indicating that NO-related mechanisms do not contribute uniformly to neurodegeneration [294,295]. This variability suggests that therapeutic modulation of the NO/NOS system should be tailored to patient-specific molecular features rather than applied in a standardized way. In this context, patient stratification may be considered for the identification of subgroups in which NO dysregulation plays a more central pathogenic role. Biomarkers associated with nitrosative stress, NOS activity, mitochondrial alterations, or NO-related metabolic pathways could support the selection of patients more prone to respond to targeted interventions. In addition, genetic variability influencing NO metabolism or antioxidant capacity may further modulate individual treatment responses. The clinical use of NO-related biomarkers in other medical fields (e.g., cardiovascular and pulmonary diseases) suggests how monitoring this pathway can lead to more precise and specific therapeutic directions [296,297]. In particular, the concept of NO delivery toward specific and isolated sites was successfully explored in cancer therapy, in which NIR (near-infrared)-responsive nanoparticles were employed to deliver NO directly to tumors, reversing multi-drug resistance and enhancing chemo- and photodynamic therapies [298,299,300]. From a translational point of view, personalized NO-targeted strategies in neurodegenerative diseases may consider approaches that allow regulated modulation of NO signaling within the CNS. In particular, brain-directed delivery systems and controlled-release formulations may help adapt treatment intensity and localization to the molecular profile of each patient [301,302]. Altogether, these considerations support the inclusion of NO/NOS modulation within a personalized medicine framework aimed at improving patient selection and therapeutic precision in neurodegenerative disorders.

## 6. Conclusions and Future Perspectives

NO and the NOS system occupy a central position in the pathogenesis of multiple neurodegenerative disorders, including AD, tauopathies, FTD, ALS, and PD. While physiological NO levels are essential for neuronal communication, synaptic plasticity, and vascular homeostasis, dysregulated NO production, particularly from iNOS and nNOS, contributes to nitrosative and oxidative stress, mitochondrial dysfunction, excitotoxicity, and ultimately neuronal death. The dual role of NO as both a neuroprotective and neurotoxic agent underscores the complexity of targeting this pathway therapeutically. A growing body of evidence supports the concept that restoring redox balance, rather than fully suppressing NO synthesis, offers the greatest therapeutic promise. Antioxidant-based interventions, whether synthetic or derived from natural sources, can neutralize ROS and RNS, limit ONOO^−^-mediated damage, preserve mitochondrial function, and help maintain BBB integrity. However, despite significant advances in preclinical models, translation to effective clinical therapies remains a challenge, largely due to limited human trials, the heterogeneity of disease mechanisms, and the need for agents capable of crossing the BBB and acting on multiple pathogenic pathways.

Future research should focus on developing multifunctional compounds that not only modulate NO/NOS activity but also support endogenous antioxidant defenses, repair mitochondrial deficits, and prevent excitotoxic injury. Such integrative approaches have the potential to slow or, ideally, halt the progression of neurodegeneration, shifting the focus from symptom management to true disease modification. Notably, individual variability in NO/NOS dysregulation and redox imbalance suggests that these strategies may not be completely effective for all patients. Integrating redox-related biomarkers with genetic factors and disease stage may help identify patients who could benefit from NO- and antioxidant-targeted therapies. In this context, therapy personalization represents a crucial step toward the improvement of the translational application of antioxidant agents in neurodegenerative diseases. By targeting the intersection of NO signaling, oxidative stress, and neuronal survival within a personalized medicine framework, the next generation of antioxidant therapies could open a new chapter in the treatment of neurodegenerative diseases.

## Figures and Tables

**Table 1:** Common and disease-specific mechanisms of NO-mediated oxidative/nitrosative stress in neurodegenerative disorders.

Mechanistic Pathway	AD	Tauopathies	ALS	PD	Common Mechanisms	Disease-Specific Mechanisms
NO overproduction (iNOS/nNOS)	↑ iNOS (astrocytes, microglia); nNOS via NMDA	↑ NO via oxidative stress context	↑ nNOS early; iNOS later	↑ nNOS (neurons), iNOS (microglia)	Upregulation of iNOS/nNOS driven by inflammation and excitotoxicity	AD: Aβ-driven glial iNOS; ALS: early neuronal nNOS; PD: dopaminergic neurons; Tauopathies: oxidative milieu.
ONOO^−^ formation	Drives Aβ toxicity	Promotes tau nitration	Enhanced by mutSOD1	Increased via mitochondrial ROS	Peroxynitrite formation as central cytotoxic mediator	AD: Aβ interaction; ALS: SOD1 redox; PD: mitochondrial ROS; Tauopathies: tau nitration.
Oxidative/nitrosative stress	Aβ–ROS–NO loop	ROS-driven tau pathology	SOD1 dysfunction	Dopamine + NO synergy	Self-amplifying ROS/RNS cycles	AD: Aβ loop; ALS: SOD1; PD: dopamine oxidation; Tauopathies: tau-driven ROS.
Protein modifications	S-nitrosylation, nitration	Tau modification	SOD1 nitration	S-nitrosylation of tyrosine hydroxylase (TH)	Protein dysfunction via S-nitrosylation and nitration	AD: synaptic proteins; ALS: SOD1; PD: TH; Tauopathies: tau.
Mitochondrial dysfunction	Energy failure	Reduced OXPHOS	Apoptosis pathways	Complex I deficit	NO-mediated inhibition of mitochondrial respiration	PD: complex I; ALS: apoptosis; AD: energy failure; Tauopathies: OXPHOS.
Excitotoxicity	Glutamate → Ca^2+^ influx	Indirect	nNOS activation	NMDA overactivation	NMDA-mediated Ca^2+^ influx activating nNOS	AD: glutamate dysregulation; ALS: motor neurons; PD: ATP loss.
Disease hallmark	Aβ, tau	Tau NFTs	SOD1, TDP-43	α-synuclein	NO contributes to protein aggregation	Disease-specific aggregates (Aβ/tau, tau, SOD1/TDP-43, α-synuclein).
Self-amplifying cycles	Aβ ↔ ROS/NO	ROS ↔ tau	NO ↔ ONOO^−^	Dopamine ↔ NO	Feed-forward degenerative loops	Disease-specific molecular drivers (Aβ, tau, SOD1, dopamine).

**Table 2:** Antioxidant/nitrosative therapeutic approaches. Summary of therapeutic strategies targeting oxidative and nitrosative stress in neurodegenerative disorders. Data updated to February 2026.

Therapeutic Approach	Agents	Mechanisms	Target	Relevance to Neurodegeneration	Clinical Development Stage	References
Synthetic NOS-modulating compounds	Amino acid amidine derivatives; urea/thiourea derivatives; aromatic amidines; cyclic amidines; Pyridinylbenzylamine derivatives	Partial NOS inhibition; controlled reduction of NO; modulation of NO synthesis	iNOS, nNOS, eNOS	Reduce pathological NO production while preserving physiological signaling	Preclinical	[255,256,257,258,259,274,275,276]
Less isoform-specific NOS inhibitors	N5-(1-iminoethyl)-L-ornithine; 4-Methylaminopyridine	Target multiple NOS isoforms (iNOS, eNOS, nNOS)	iNOS, eNOS, nNOS	Wider neuroprotection across NO-related pathways	Preclinical	[256,268,269,271,277]
Natural/nutraceutical antioxidants	Polyphenols; flavonoids; carnosine; plant-derived bioactives	ROS scavenging; reduction of ONOO^−^; restoration of redox homeostasis	Mitochondrial and cytosolic ROS/RNS	Indirect regulation of NO/NOS balance; neuronal survival	Preclinical/early clinical evidence	[259,260,261,262,265,278,279]
Indirect antioxidant mechanisms	Glutathione replenishment; BBB preservation; mitochondrial enhancement	Strengthening endogenous antioxidant defenses; stabilization of neuronal environment	BBB integrity, mitochondria, redox buffering	Prevents vicious cycle of oxidative/nitrosative stress driving neuronal death in AD, tauopathies, FTD, and PD	Preclinical	[269,270,271]
Clinical translation	—	—	Oxidative/nitrosative stress pathways	No approved NOS-targeting therapies currently available	No approved therapies	[256,272,273]

## Data Availability

The information supporting this study’s findings is available in this article. No new data were created or analyzed in this study. Data sharing is not applicable to this article.

## Abbreviations

The following abbreviations are used in this manuscript:

Aβ	Amyloid-beta
AD	Alzheimer’s disease
ALDH	Aldehyde dehydrogenase
ALS	Amyotrophic lateral sclerosis
APP	Amyloid precursor protein
BBB	Blood–brain barrier
BDNF	Brain-derived neurotrophic factor
CA1/CA3	Cornu ammonis region 1/3
CAT1	Cationic amino acid transporter 1
CDK5	Cyclin-dependent kinase 5
cGMP	Cyclic guanosine monophosphate
CREB	cAMP response element-binding protein
CNS	Central nervous system
C9orf72	Chromosome 9 open reading frame 72
DOPAC	3,4-Dihydroxyphenylacetic acid
eNOS	Endothelial nitric oxide synthase
ER	Endoplasmic reticulum
fALS	Familial ALS
FUS	Fused in sarcoma (RNA-binding protein)
FTD	Frontotemporal dementia
FTLD	Frontotemporal lobar degeneration
FTDP-17	Frontotemporal dementia with parkinsonism linked to chromosome 17
GSH	Glutathione
GPx	Glutathione peroxidase
GRN	Progranulin gene
GSK-3β	Glycogen synthase kinase 3 beta
iNOS	Inducible nitric oxide synthase
JNK	c-Jun N-terminal kinase
LTP	Long-term potentiation
MAO	Monoamine oxidase
MAP	Microtubule-associated protein
MAPT	Microtubule-associated protein tau gene
MDA	Malondialdehyde
Mn-SOD	Manganese superoxide dismutase
MT	Microtubule
mtNOS	Mitochondrial nitric oxide synthase
NADPH	Nicotinamide adenine dinucleotide phosphate (reduced form)
NFTs	Neurofibrillary tangles
NMDA/NMDAR	N-methyl-D-aspartate receptor
NO	Nitric oxide
NOS	Nitric oxide synthase
NOS1/nNOS	Neuronal nitric oxide synthase
NOS2/iNOS	Inducible nitric oxide synthase
NOS3/eNOS	Endothelial nitric oxide synthase
NOS1AP	Nitric oxide synthase 1 adaptor protein
ONOO^−^	Peroxynitrite
OS	Oxidative stress
PARP	Poly (ADP-ribose) polymerase
PD	Parkinson’s disease
PNS	Peripheral nervous system
PP2A	Protein phosphatase 2A
PTP	Permeability transition pore
RNS	Reactive nitrogen species
ROS	Reactive oxygen species
sALS	Sporadic ALS
SNP	Sodium nitroprusside
SNO	S-nitrosylated/S-nitrosylation
SNpc	Substantia nigra pars compacta
SOD1	Superoxide dismutase 1
Sp1	Specificity protein 1 (transcription factor)
TDP-43/TARDBP	TAR DNA-binding protein 43
